# Characterizing the species composition of European Culicoides vectors by means of the Köppen-Geiger climate classification

**DOI:** 10.1186/1756-3305-6-333

**Published:** 2013-11-22

**Authors:** Katharina Brugger, Franz Rubel

**Affiliations:** 1 Institute for Veterinary Public Health, University of Veterinary Medicine Vienna, Veterinärplatz 1, 1210, Vienna, Austria

**Keywords:** Bluetongue, *Culicoides obsoletus* complex, *C. pulicaris*, *C. imicola*, Climate

## Abstract

**Background:**

Biting midges of the genus *Culicoides spp.* (Diptera: *Ceratopogonidae*) are vectors for the Bluetongue virus, the African horse sickness virus and the recently emerged Schmallenberg virus. Here, species of the *C. obsoletus* complex, the *C. pulicaris* complex and *C. imicola* were considered. The objective was to compile a map of these *Culicoides* species and their relation to the popular climate classification defined by Wladimir Köppen and Rudolf Geiger to provide a quick view on the species composition in Europe.

**Findings:**

Major parts of Central and Northern Europe are covered by a warm temperate fully humid climate, characterized by warm summers. For this so-called Cfb climate fractions of 89% *C. obsoletus* complex and 11% *C. pulicaris* complex were estimated. Further investigations comprise the continental climate Dfb (76% *C. obsoletus*, 24% *C. pulicaris*), the warm temperate climate with hot summers Cfa (35% *C. obsoletus*, 65% *C. pulicaris*), the warm temperate dry climate, characterized by warm summers Csb (38% *C. obsoletus*, 51% *C. pulicaris*, 11% *C. imicola*) and the warm temperate dry climate with hot summers Csa of the Mediterranean area (11% *C. obsoletus*, 12% *C. pulicaris*, 77% *C. imicola*).

**Conclusions:**

A highly significant association coefficient of R_V_ = 0.64 (Cramer’s V) confirms the correlation between *Culicoides spp.* and climate zones. Moreover, climate projections for the end of the century give an impression on expected changes in the European *Culicoides spp.* composition.

## Background

Within the past few years *Culicoides* species compositions were investigated in many national studies comprising different climate regions. However, a generalised map for Europe is missing. Such a map may be helpful especially for non-entomologists who want to get a quick view on *Culicoides* vectors. Additionally, experts get a first qualitative impression on the species composition expected for different climate change scenarios. To compile such a map, the authors used their own monitoring data [1] as well as data from the literature [2-13]. In contrast to the detailed national studies mentioned above, a large-scale analysis is presented here. To obtain a clear statistical signal for the *big picture* all details were removed from the data by averaging. The focus is on the main Bluetongue vector species, those of the *C. obsoletus* complex, the *C. pulicaris* complex and *C. imicola*[14]. It is further known that midges of the *C. obsoletus* complex are most abundant in the fully humid climates of Central and Northern Europe, while *C. imicola* are the main vectors for the Bluetongue virus in the dry Mediterranean area. A North-south orientated gradient in the species composition is predominant. Quantitative relationships to climate zones, however, are still missing. So far *Culicoides* distribution maps are so-called presence/absence maps as recently presented by e.g. [15] or maps depicting the density of the main Bluetongue vectors [1].

**Figure 1:**
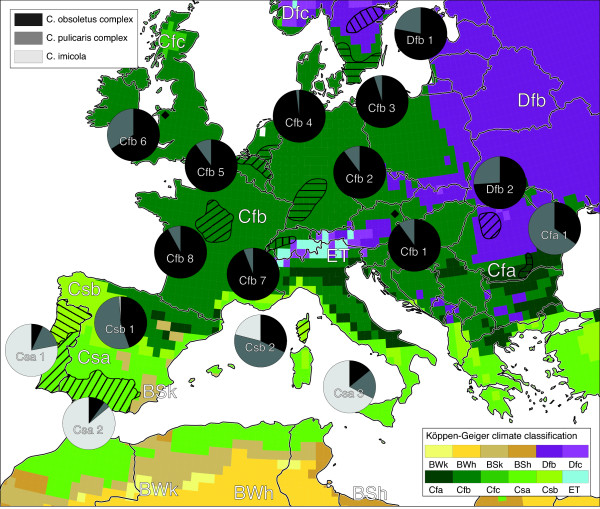
**Spatial distribution of observed *Culicoides spp. *compositions underlaid with a map of the Köppen-Geiger climate classification.** Monitoring regions were marked by shaded areas and squares for which pie charts were compiled. Pie chart labels refer to the climate class and the study referenced in Table 1.

Here, this gap is closed by relating the 3 species complexes to 5 climate classes of the warm temperate and continental climates defined by the well-known Köppen-Geiger climate classification [16]. A digital version of this climate classification was compiled by [17] and projected to the future by [18]. It is publicly available on a 0.5° regular longitudinal/latitudinal grid (http://koeppen-geiger.vu-wien.ac.at). Each climate is characterized by means of temperature and precipitation described by a three-letter code as documented by [17]. Because *Culicoides spp.* are the main vectors of the Bluetongue virus, which is observed almost exclusively in warm temperate and continental climates, we focus on these main climates. The warm temperate climate is denoted by the first letter C, the continental climate by D. The second letter distinguishes between humid (f) and dry (s) conditions and the third letter between warm (b) and hot (a) summers. Thus, the 5 European climate classes considered in this study are Cfb, Dfb, Cfa, Csb and Csa, respectively (Figure 1).

## Findings

Although many *Culicoides spp.* monitoring programs have been undertaken in recent years, only some of them provide unambiguous information on the species composition related to specific climate zones. Data from these monitoring programs, conducted between 2000 and 2011, were summarized in Table 1. Unless the *Culicoides* counts were not pooled by the authors of these studies, they were grouped according to [19] into *Culicoides obsoletus* complex (comprising *C. obsoletus*, *C. scoticus*, *C. dewulfi*, and *C. chiopterus*), *Culicoides pulicaris* complex (comprising *C. pulicaris*, *C. impunctatus*, *C. punctatus*, *C. grisescens*, and *C. newsteadi*), *Culicoides imicola* and other *Culicoides* species. For a clear representation the latter were excluded from the statistics. The fractions of the three species complexes were calculated separately for each climate zone and each study is referenced in Table 1, respectively. Finally, the mean fractions calculated from all studies are summarized in Table 2. Note, that the sum of fractions in each climate zone is 1 (100%).

**Table 1:** ***Culicoides spp. *** **monitoring data for different climate zones used in this study**

**Climate**	**Country**	**Number of traps**	**Type of traps**^**1**^	**Sampling period**	**Sampling interval**	**Reference**
Cfb 1	AT	1	OVI	2009-2011	Daily	[1]
Cfb 2	DE	9	BG	2007-2008	Day 1-8 of each month	[2]
Cfb 3	SE	12	OVI	2008-2009	Weekly	[3]
Cfb 4	NL	4	LP	2005-2006	Continuously and emptied once a week	[4]
Cfb 5	BE	29	CDC	2006	Two nights twice a week	[5]
Cfb 6	UK	35	OVI	2008	Three trapping nights	[6]
Cfb 7	CH	4	OVI	2008-2011	Weekly	[7]
Cfb 8	FR	n/a	OVI	2010	Weekly	[8]
Dfb 1	SE	2	OVI	2008-2009	Weekly	[3]
Dfb 2	RO	3	OVI	2008	Weekly in May	[9]
Cfa 1	RO	3	OVI	2008	Weekly in May	[9]
Csb 1	PT	35	OVI	2000/2001	Two to seven consecutive nights	[10]
Csb 2	FR	12	OVI	2002	One night every three weeks	[11]
Csa 1	PT	43	OVI	2000/2001	Two to seven consecutive nights	[10]
Csa 2	ES	52	CDC	2007-2008	Weekly	[12]
Csa 3	IT	n/a	OVI	2000-2003	Weekly	[13]

^1^OVI - Onderstepoort Veterinary Institute black light suction trap; CDC - new standard miniature black light trap (model 1212), John W. Hook Company, USA; BG - BG-Sentinel mosquito trap, Biogents AG, Germany; LP - Liberty Plus CO_2_ baited counterflow trap, America Biophysics Co., USA.

**Table 2:** **Contingency table for *Culicoides spp. *fractions related to different climate zones resulting in an association coefficient (Cramer’s V) of R**_**V**_ **= 0.64**

**Climate**	***Culicoides *** **species complex**			
	***obsoletus***	***pulicaris***	***imicola***	
Cfb (warm temperate, moist, warm summer)	0.89	0.11	0.00	1.00
Dfb (continental, moist, warm summer)	0.76	0.24	0.00	1.00
Cfa (warm temperate, moist, hot summer)	0.35	0.65	0.00	1.00
Csb (warm temperate, dry, warm summer)	0.38	0.51	0.11	1.00
Csa (warm temperate, dry, hot summer)	0.11	0.12	0.77	1.00
	2.49	1.63	0.88	5.00

Figure 1 provides a quick view on the composition of European *Culicoides spp.* and their relation to climate. Major parts of Central and Northern Europe are covered by a warm temperate (green areas) and a continental or snow (violet areas) climate for which the *Culicoides spp.* compositions were investigated. The fully humid climates characterized by warm summers are denoted by Cfb and Dfb. The typical species composition of Cfb climate comprises 89% midges of the *C. obsoletus* complex and 11% of the *C. pulicaris* complex. For the Dfb climate 76% *C. obsoletus* and 24% *C. pulicaris* were estimated. No *C. imicola* were observed in Cfb and Dfb climates. The similar climate zone with hot summers is denoted by Cfa, for which on average 35% *C. obsoletus* and 65% *C. pulicaris* were found. The Csb climate, the warm temperate dry climate characterized by warm summers, is mainly observed in Northern Spain and Portugal, but also in the inland of Greece and Italy. It is characterized by a species composition of 38% *C. obsoletus*, 51% *C. pulicaris* and 11% *C. imicola*. For the dry climate with hot summers, usually known as Mediterranean or Csa climate, a mean fraction of 11% *C. obsoletus*, 12% *C. pulicaris* and 77% *C. imicola* was observed (Table 2). The association coefficient of R_V_ = 0.64 (Cramer’s V) confirms the correlation between *Culicoides spp.* and climate zones. Because of the huge sampling size this correlation is highly significant (p < 0.01). Note that the latter may not be calculated from Table 2 where - for a better representation - fractions instead of absolute numbers were presented. Generally, the fractions of *C. obsoletus* decrease while those of *C. imicola* increase from Cfb to Csa climate (North-south gradient). The fraction of *C. pulicaris* is maximal in Cfa and Csb climate.

## Conclusions

A quick view on the composition of the most important *Culicoides spp.* in Europe was introduced. To the knowledge of the authors this is the first map on this topic. The results are widely consistent with previously published maps such as the possible distribution of *C. imicola* in Spain [15], the presence/absence maps of *C. imicola* around the Mediterranean area predicted from sites in Portugal [20] and the Iberian Peninsula [21] or the northern and southern distribution limits for *C. obsoletus*, *C. pulicaris* and *C. imicola* groups [22].

Although a significant correlation between *Culicoides spp.* composition and climate zones was demonstrated, the spatial resolution applied is rather coarse and may be refined by using future higher resolution climate maps and advanced statistical approaches such as niche modeling (e.g. [23]). A further weak point of the study is that, due to the lack of other data sources, *Culicoides* counts from different trapping methods were mixed together. These comprise various black light suction trap types and a CO_2_ baited counterflow trap type (Table 1). More consistent results may be obtained by using only one standardized trapping method. Improved analyses should also be based on extensive data to be compiled from national sources as currently conducted within the EMIDA VICE project (Carsten Kirkeby, pers. communication).

**Figure 2:**
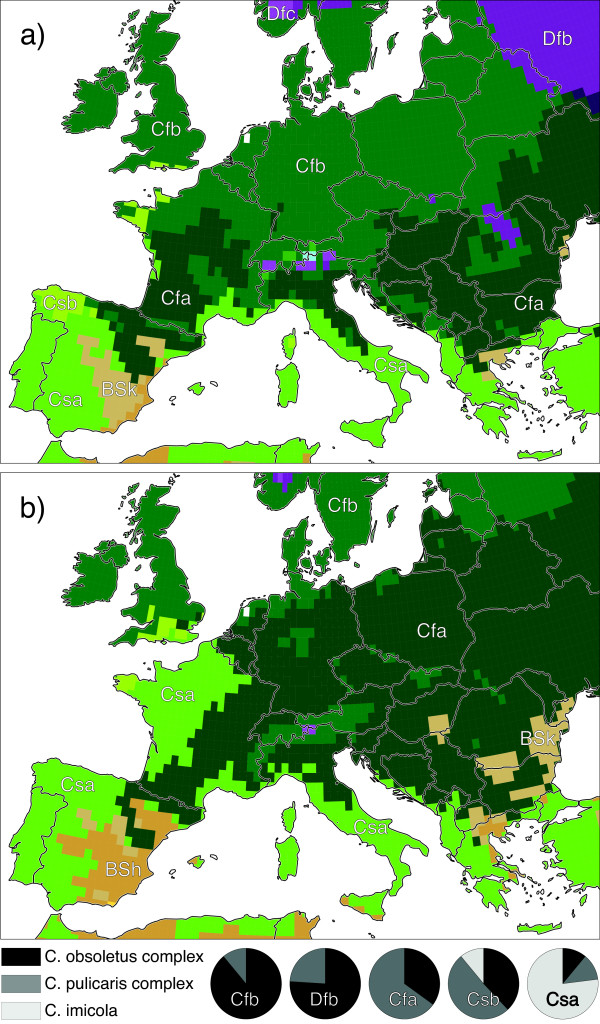
**Projected Köppen-Geiger climate classifications for the best-case (a) and the worst-case (b) IPCC emission scenario.** Period 2076–2100, after [18].

Finally, Figure 2 may be used to compare current (Figure 1) with expected *Culicoides spp.* compositions (Table 2) by means of Köppen-Geiger climate classifications for the period 2076-2100 [18]. The projections were compiled from multi-model ensemble predictions of temperature and precipitation following the Intergovernmental Panel on Climate Change (IPCC) emission scenarios B1 (minor climate changes, called best-case scenario) and A1FI (major climate changes, called worst-case scenario).

After both scenarios the warm temperate climate shifts to the North and East, in which large areas covered by the fully humid climate with warm summers (Cfb) will be replaced by the humid climate with hot summers (Cfa). Thus the future species composition in Central and Northern Europe will be characterized by a decreasing fraction of *C. obsoletus* and an increasing fraction of *C. pulicaris*. Another example concerns the dry climates Csb and Csa, which are projected to extend their ranges from Spain to Northern France resulting in a northward shift of the distribution of *C. imicola*. In arid regions, projected by the worst-case IPPC scenario for the south of the Iberian Peninsula and the Thessaloniki region in Greece (BSk and BSh climates in Figure 2), however, environmental conditions for *C. imicola* will worsen. As [24] stated soil moisture may be considered as a key environmental variable for the delineation of suitable *C. imicola* habitat. Thus, climate projections for the end of the century give an impression on expected changes of the *Culicoides spp.* composition. A final statement on epidemiological consequences concerning the shift in *Culicoides spp.* compositions, however, is rather speculative. As *C. imicola* is a confirmed field vector for the African horse sickness virus [25] and the BTV serotypes 1, 2, 4, 6 and 9 [22], upcoming outbreaks might be occur in regions covered by Csa and Csb climates in future decades. On the other hand, changing vector competences (e.g. shorter extrinsic incubation periods due to global warming) and new virus serotypes may still lead to unforeseeable disease outbreaks. As an example for the latter, the BTV-8 outbreak in Central Europe 2006 is mentioned. It took place in Cfb climates, a region where *C. imicola* is absent.

## Competing interests

The authors declare that they have no competing interests.

## Author’s contributions

The project was designed, the data analysed and the paper written by KB and FR. Both authors read and approved the final version of the manuscript.
